# The condition‐dependence of male genital size and shape

**DOI:** 10.1002/ece3.11180

**Published:** 2024-03-17

**Authors:** Tanya M. Pennell, Manmohan D. Sharma, Andreas Sutter, Drew T. Wilson, Clarissa M. House, David J. Hosken

**Affiliations:** ^1^ Centre for Ecology & Conservation, Faculty of Environment, Science and Economy (ESE) University of Exeter Exeter UK; ^2^ School of Science Western Sydney University Richmond New South Wales Australia

**Keywords:** condition, diet, *Drosophila*, genitals, inbreeding depression, sexual selection

## Abstract

The male genitals of internal fertilisers evolve rapidly and divergently, and sexual selection is generally responsible for this. Many sexually selected traits are condition‐dependent—with their expression dependent upon the resources available to be allocated to them—as revealed by genetic or environmental manipulations of condition. However, it is not clear whether male genitals are also condition‐dependent. Here we manipulate condition in two ways (via inbreeding and diet) to test the condition‐dependence of the genital arch of *Drosophila simulans*. We found that genital size but not genital shape suffered from inbreeding depression, whereas genital size and shape were affected by dietary manipulation of condition. The differential effects of these treatments likely reflect underlying genetic architecture that has been shaped by past selection: inbreeding depression is only expected when traits have a history of directional selection, while diet impacts traits regardless of historical selection. Nonetheless, our results suggest genitals can be condition‐dependent like other sexually selected traits.

## Graphical abstract

Genital size and shape are condition‐dependent: both traits are impacted by diet, but only size is affected by genetic condition (inbreeding). The contrasting effects of inbreeding on these two traits likely reflect underlying genetic architecture that has been shaped by past selection. Overall, our results suggest that male genitals can be condition‐dependent much like other sexual characters.

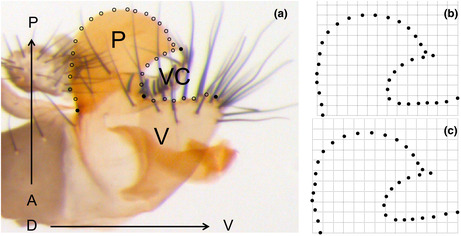

## 1 INTRODUCTION

Sexual selection is responsible for rapid, divergent evolution, and low intraspecies variation in the male genitals of internally fertilising species (Arnqvist et al., 1997; Eberhard, 1985, 2009; Eberhard et al., 1998; Hosken & Stockley, 2004). Furthermore, male genitals should be closely linked to fitness given their fundamental role in reproduction, and many studies have now formally linked genital form to fertilisation success (e.g. Arnqvist & Danielsson, 1999; Gasparini et al., 2011; House et al., 2021; House & Simmons, 2003; Wojcieszek & Simmons, 2011), a key male fitness component.

Characters closely linked to fitness, including many sexually selected traits, are frequently condition‐dependent, with their expression dependent upon the pool of resources that are available to be allocated to them (Rowe & Houle, 1996). Condition dependence evolves because these traits are relatively costly to develop and/or maintain. Condition is analogous to ‘residual reproductive value’ (the expected amount of reproductive output remaining in an individual's life) or ‘state’ (individual attributes like fat/protein reserves, or foraging skills: McNamara & Houston, 1996) in life‐history models (Rowe & Houle, 1996) and variation in condition can have a genetic basis and/or arise from environmental factors (Tomkins et al., 2004). For example, allelic variation at various loci is likely to influence resource acquisition and consequently the expression of costly traits (Rowe & Houle, 1996), as will the quantity and quality of resources harvested from the environment (Tomkins et al., 2004). Additionally, condition‐dependent trait expression is important in maintaining honesty in sexual signalling which, for example, enables females to select high‐quality males as mates (Rowe & Houle, 1996).

While individual condition can be difficult to measure, it can be manipulated both genetically and environmentally (e.g. Bonduriansky et al., 2015; Bradbury & Blakey, 1998; David et al., 2000; Hooper & Bonduriansky, 2022; House et al., 2016; Hunt et al., 2005; Prokop et al., 2010). A primary way to manipulate condition environmentally is through diet, to directly control the resources that are available to an individual (Droney, 1998; Ketola & Kotiaho, 2010): individuals on poor diets will be in poorer condition as they have less resource available to allocate to fitness enhancement (e.g. Bradbury & Blakey, 1998; David et al., 2000; Hooper & Bonduriansky, 2022; House et al., 2016). Similarly, inbreeding can be used to manipulate condition genetically (Bellamy et al., 2014; Tomkins et al., 2004). Inbreeding exposes deleterious recessive alleles, or leads to the loss of high fitness heterozygotes, and both mechanisms may result in inbreeding depression (loss of fitness), although deleterious recessive exposure seems to be the more general explanation for fitness reductions (Lynch & Walsh, 1998). Regardless of the mechanism, more inbred individuals tend to be of lower quality, being less able to accumulate, assimilate or allocate resources. As a result, inbred individuals can be viewed as being in poorer genetic condition (e.g. Keller et al., 1994), and there have been calls for more investigation into genetic condition dependence (Bellamy et al., 2014; Tomkins et al., 2004). Thus, inbreeding and diet are seen to be effective ways to manipulate condition and test for condition dependence (Bellamy et al., 2014).

Manipulation of condition via inbreeding also offers insights beyond condition dependence. The magnitude of fitness loss due to inbreeding depends on the directional dominance underlying a trait (Falconer, 1989; Lynch & Walsh, 1998). Directional dominance describes dominance that is biased in one direction (e.g. toward smaller size) when summed across loci. It is predicted to result from the selective removal of dominant deleterious alleles, coupled with the fixation of additive beneficial alleles and retention of deleterious recessives that are largely hidden from selection (Falconer, 1989; Lynch & Walsh, 1998). Hidden deleterious recessives can be revealed by inbreeding, causing the trait value to move in the direction of low fitness. Traits with a history of directional selection have relatively high directional dominance and therefore tend to show greater inbreeding depression (Charlesworth & Charlesworth, 1987, 1999; Lynch & Walsh, 1998; Roff, 2002). Conversely, traits under stabilising selection (or those weakly associated with fitness) have lower levels of directional dominance and show little or no inbreeding depression (Lynch & Walsh, 1998). Thus, inbreeding depression or its absence can also be used to infer whether selection has historically been stabilising or directional, noting again that inbreeding depression is always in the direction of low fitness (e.g. Ala‐Honkola et al., 2013; Ketola & Kotiaho, 2012).

While there is much evidence for the condition dependence of sexually selected traits (Arnqvist et al., 1997; Arnqvist & Thornhill, 1998; Tomkins et al., 2004), little is known about condition effects on genitals (e.g. Knell & Simmons, 2010; Rogers et al., 2008), although other sexual traits involved directly in reproduction, such as sperm morphology and motility, display inbreeding depression (Asa et al., 2007; Fitzpatrick & Evans, 2009; Gage et al., 2006; Gomendio et al., 2000) and are therefore condition‐dependent. However, it is unclear if genitals are similarly impacted by inbreeding. On the one hand, male genitals may show inbreeding depression because a history of rapid divergence (e.g. Eberhard, 1985) may be indicative of past directional selection and more directional dominance (see above), but it has also been suggested that male genital form is under stabilising selection. Stabilising selection could be generated by the need to fit the average sized female in a population (the one‐size‐fits‐all hypothesis; Eberhard et al., 1998; and see Wojcieszek & Simmons, 2012). This inference is supported by evidence of male–female genital coevolution (e.g. Kamimura & Mitsumoto, 2011; Yassin & Orgogozo, 2013; but see Jagadeeshan & Singh, 2006) and negative genital allometry in males (Eberhard et al., 1998; Hosken et al., 2005).

It is also unclear if male genital form is influenced by environment‐induced condition, with the few studies testing this producing mixed results (Arnqvist & Thornhill, 1998; Cayetano & Bonduriansky, 2015; House & Simmons, 2007; Shingleton et al., 2009; Wylde & Bonduriansky, 2020). If genital evolution is determined by the need for male and female genitals to match precisely, then genitals are expected to be relatively canalised and robust to environmental stress (Arnqvist, 1997). Indeed, canalisation may explain the low intraspecies variation seen in genital form (e.g. Eberhard, 1985). Thus, genitals may display limited environment‐induced condition dependence. In contrast, environment‐induced condition dependence may be high if sexual selection favours exaggerated genital traits that are developmentally costly. Environment‐induced condition dependence may also be revealed in relatively canalised traits if, for example, individuals are exposed to conditions outside of their typical range. Lastly, inbreeding may affect traits in similar ways to diet, through its impacts on resource acquisition and assimilation (Rowe & Houle, 1996), but this may only be apparent in resource‐restricted environments (see Hooper & Bonduriansky, 2022).

Overall, it is unclear if male genital morphology is condition‐dependent and there have been few investigations of this. Here we tested for the condition dependence of the size and shape of the posterior and ventral lobes of the *Drosophila simulans* genital arch, using a diet manipulation to alter environment‐induced condition and one generation of full‐sib mating as our inbreeding (genetic) manipulation of condition. Genital traits in *D. simiulans* are under strong selection (House et al., 2013, 2021), and there is clear interspecific variation in genital arch morphology between *D. simulans* and its close relatives *D. melanogaster* and *D. mauritiana* (Coyne, 1983). Moreover, the genital arch is an extension of the epandrium and is functionally important for grasping female genitals (Jagadeeshan & Singh, 2006). This morphological structure is thought to be important during ejaculate transfer as it allows male and female genitals to ‘mesh’ (Eberhard & Ramirez, 2004). Following Eberhard's (1985) definition of male genitals – structures associated with the gonopore that interact with (including holding the female in precise ways) the female during copulation – the genital arch is an integral component of the male genitals of *D. simulans*. Although genital form variation affects fitness in *D. simulans* (House et al., 2013, 2021), condition dependence remains difficult to predict for reasons noted above.

In addition to assessing the condition dependence of genitals, we use inbreeding as an indicator of the form of past selection likely to have acted on the genital arch. As we use an ad‐libitum diet in our inbreeding experiment, any trait responses to this treatment most likely reflect underlying genetic architecture, as opposed to more general effects of inbreeding on resource allocation. The response of genitals to inbreeding is difficult to predict, as genital components differ in selective histories and degree of directional dominance. For example, the genital arch seems to have undergone directional selection in its recent evolutionary history (Zeng et al., 2000) and may currently be under directional selection, with both natural and sexual selection favouring similar trait values (House et al., 2013). However, some components of *D. simulans* genital form are under stabilising selection (House et al., 2013, 2021).

We also compare the effects of both inbreeding and diet on genital morphology to that of leg size, a non‐sexual trait that reflects body size and is assumed to be under stabilising selection in *Drosophila* (e.g. Mackay, 2010). We expect leg size will display diet‐induced condition dependence, as growth and body size in this species is affected by resource availability (e.g. Blanckenhorn, 1999; Dmitriew, 2011). On the other hand, stabilising selection on body size (e.g. Mackay, 2010) should mean low directional dominance and a lack of inbreeding depression.

## 2 MATERIALS AND METHODS

### 2.1 Derivation of fly stocks

The *D. simulans* stock population was derived from flies collected in Australia in 2004 and reared as a large, outbred population (ca. 1000 individuals) at 25°C using a 12/12 h light/dark cycle since 2006. The population harbours ample phenotypic and genetic variation for every trait examined so far (e.g. Hosken et al., 2008; Okada et al., 2011; Sharma et al., 2010, 2012; Taylor et al., 2008; Wright et al., 2008). The flies used here were reared on standard *Drosophila* diets – Jazz Mix (Fisher Scientific UK Ltd, Loughborough, Leicestershire, UK) and porridge mix (see below) – the use of which have no differential effects on fly size or fitness. Ice and carbon dioxide anaesthesia were used for handling and transferring flies. Unless stated otherwise, the virgin flies used throughout both experiments were 3–4 day old and collected within 6 h of eclosion (to ensure virginity).

### 2.2 Inbreeding manipulation

**FIGURE 1:**
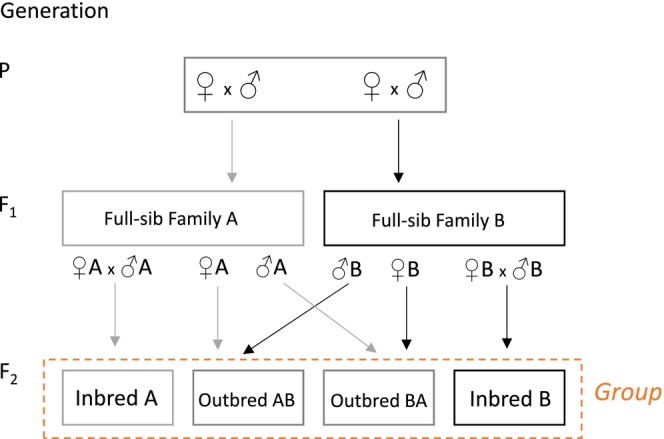
Roff's (1998) crossing design used to generate inbred and outbred experimental F2 offspring shown for a single “group”. Each group produces two F2 inbred lines through F1 brother–sister matings and two F2 outbred lines from reciprocal crosses between the two F1 families. This design minimises the likelihood of different allelic representation in inbred and outbred individuals.

Replicate inbred and outbred lines were generated (Figure 1) using the Roff (1998) crossing design (also see Okada et al., 2011; Wright et al., 2008). In principle, this design leads to equal representation of alleles in the inbred and outbred groups (Roff, 1998). As detailed below, we employed one generation of inbreeding. Previous work by us has shown that this is sufficient to cause inbreeding depression in a range of traits from morphology to life‐history (Okada et al., 2011; Wright et al., 2008). Importantly, male fertility (a key male fitness component) is reduced at this level of inbreeding (Okada et al., 2011), showing that male condition is reduced in the offspring of the sib‐sib matings we use here. Moreover, this design avoids the possibility of purging and a recovery of fitness which may be associated with successive generations of inbreeding (Crnokrak & Barrett, 2002).

Briefly, laying vials were collected from stock population cages and the adults which eclosed from these formed the parental generation (P). P‐flies were collected as 3‐day‐old virgin flies and haphazardly paired in individual vials to produce F1 full‐sib families (F1; *N* = 120). To produce in‐ and outbred animals we randomly allocated F1 families to groups, so that each group consisted of two‐unrelated full‐sib families (this should have resulted in 60 groups, but we finally had 53 groups made up of pairs of families). Virgin F1 flies from each full‐sib family were then collected and crossed to create inbred and outbred F2 flies within each group (i.e. there were within‐family and across‐family, within‐group crosses: Figure 1). The inbred flies (theoretical *F* = 0.25) resulted from mating between brothers and sisters (which again is sufficient to cause inbreeding depression: Okada et al., 2011; Wright et al., 2008), whereas the outbred flies were produced by reciprocally crossing a male and a female from different families within each two‐family group. Subsequently, inbred and outbred flies were dissected for morphometric analyses. Vials containing developing F2 flies were incubated for 7 days after the first F2 adult eclosed. This period allows the majority of F2 offspring to eclose but prevents completion of the development of any offspring from possible F2 matings. Vials were then frozen at −20°C. For each inbred/outbred F2 family at least three males were haphazardly selected for dissection (inbred: mean *n* = 4.87, SE = 0.053, total *n* = 487; outbred: mean *n* = 4.89, SE = 0.058, total *n* = 489) (see Section 2.4, below).

### 2.3 Diet manipulation

Parents were collected from stock‐cages as above. Virgin male–female parental pairs were randomly allocated to either a food restriction (40% diet restriction) or food ad‐libitum diet (see Table S1). Two hundred male–female parental pairs were set up in individual culture vials for each treatment. The offspring produced therefore developed under either a restricted or ad‐libitum diet. This is a crucial phase of development in *Drosophila*, and other holometabolous insects, as the size of structural traits in adults (such as body size) is determined during larval stages (Ashburner, 1989; Bakker, 1959). After the first eclosion, vials were incubated for 7 days (as above) and then a sample of male F1 offspring were frozen at −20°C for dissection.

To test for an effect of diet restriction on condition, a subset of F1 virgin males (*n* = 30) from each treatment was provided with five virgin, stock females every 24 h for 3 days (15 females/male with males moved to a new vial every day). Females were allowed to oviposit for 48 h and then moved to a second vial for another 48 h before being killed. The F1 males used for the fitness assay were frozen at −20°C after their third female set. Male fitness was measured as total offspring sired across all 15 females. Our method provided an upper‐limit to male fitness, as mating occurred in a non‐competitive environment. From each diet treatment, 125 frozen males from 125 vials (1 male per vial) were haphazardly selected for dissection (=1 male/pair, but not all pairs contributed to the final dataset). It should be noted that our design was not intended to assess allocation and investment in current versus future reproduction in response to diet.

### 2.4 Dissection and morphometrics

**FIGURE 2:**
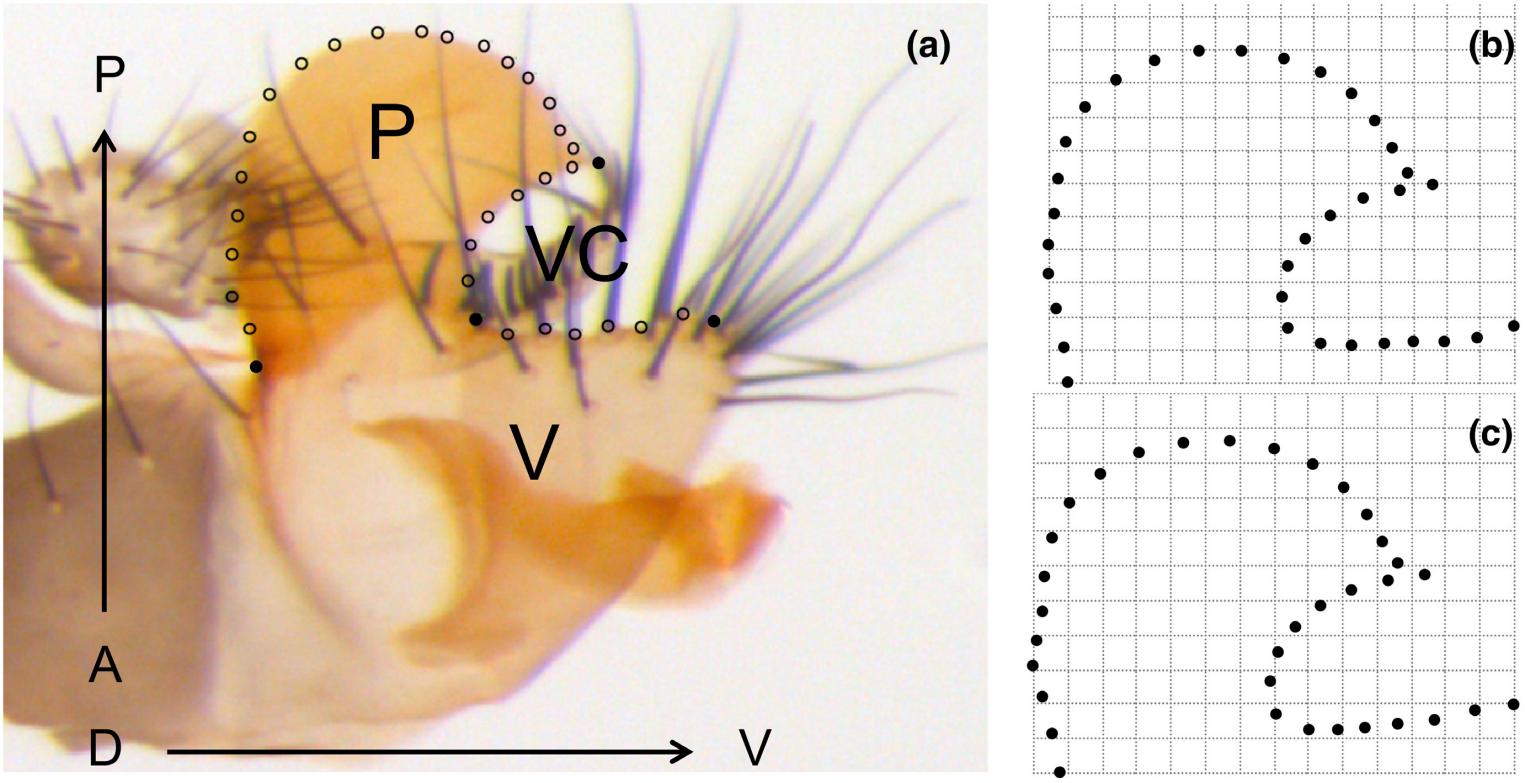
Genital arch morphometrics: (a) *D. simulans* genital arch showing the posterior lobe (P), the ventral lobe (V) and the ventral cup (VC). Changes in shape are defined by movement along the anterior–posterior (A & P) and the dorso‐ventral (D & V) axis. The filled black points are the type‐two landmarks (*n* = 4) and the open points represent the sliding landmarks (*n* = 30). (b, c) show the consensus configuration for the inbreeding and diet manipulation experiments, respectively. The 34 landmarks yield 64 relative warps (RWs) that each explain less variance than the preceding RW.

All male genitals were dissected in a drop of 50:50 glycerol and lactic acid, which softens the tissue. Genital arches were separated, oriented consistently, and mounted using Hoyer's medium. One genital arch was haphazardly chosen for imaging and images were captured using a Leica M125 microscope with a mounted camera. Geometric morphometric analyses were used to quantify the variation in size and shape of the posterior and ventral lobe of the genital arch. Four type‐two landmarks (based on geometric criteria) and 30 sliding semi‐landmarks (which can slide along the outline to minimise shape change between specimens and the Procrustes average fit) were applied across all specimens (Rohlf, 2010a; Figure 2). All images were collated using TPSUtil v1.49 (Rohlf, 2012) and all points (landmarks and semi‐landmarks) were digitised in TPSDig2 v2.16 (Rohlf, 2010b). Semi‐landmarks were identified by a ‘slider file’ generated in TPSUtil v1.49. Non‐shape variation was removed by normalising landmark data for scale, orientation, and position (Procrustes Superimposition, see Dryden & Mardia, 1998; Goodall, 1991). From these data scaled centroid size (the square root of all the landmarks summed distance from the centroid) was extracted and the variation in shape was reduced to a series of relative warp (RW) scores. Analyses from both experiments produced 64 RWs' with each sequential warp explaining less variance than its predecessor. Each of RWs 1–5 individually explained more than 5% of the shape variance and were retained for further analyses (Zelditch et al., 2004; Table 1). We also took measurements of leg length (hind tibia length: cf House et al., 2013) as an index of body size: the left or right hind tibia was haphazardly selected, removed from the thorax and mounted using Hoyer's medium. Digital images of the hind tibia were captured using a Leica M125 microscope.

**TABLE 1:** Cumulative variation in shape explained by successive relative warps (RW) 1–5 in the inbreeding and diet manipulations.

	RW 1	RW 2	RW 3	RW 4	RW 5	Total
Inbreeding manipulation	34.03%	17.67%	11.25%	8.59%	5.11%	76.65%
Diet manipulation	35.57%	22.26%	8.51%	5.75%	5.22%	77.31%

To assess measurement error, we digitised a sample of 25 images twice and measured centroid size and RW 1–5 for each image. We measured the strength of agreement between the two measurements using the Intraclass Correlation Coefficient (package ‘irr’ in R version 3.6.3: Gamer et al., 2012). Analyses confirmed that these techniques were highly repeatable (Centroid size: κ = 0.98 and *p* < .001; RW 1–5: kappa values ranged from 0.88 to 0.99 and all *p* < .001). Note that the RWs were extracted for each experiment (inbreeding or diet) separately and these values were therefore not directly comparable. Repeatability analysis was not conducted for leg length measurement, but a previous study has shown high repeatability using the same technique (House et al., 2013).

### 2.5 Statistical analysis

To test whether diet restriction reduced male condition, we used a generalised linear model (Poisson error structure), with total offspring count as the dependent variable and diet treatment as the predictor. The effects of inbreeding and diet on scaled centroid size (genital arch size) and leg length (our index of body size) was examined using univariate analysis of variance (ANOVA: type III sums of squares) and effects on shape (RW 1–5 scores) was examined using multivariate analyses (MANOVA: type III sums of squares). Inbreeding status (inbred/outbred) and diet (high/low fitness) were predictor variables in respective analyses. We included leg length as a covariate in analyses of scaled centroid size to remove variance in scaled centroid size that is due to variation in body size. Additionally, scaled centroid size and leg length were included as covariates in all shape analyses to remove any variance in shape that is associated with size. Shape changes are often associated with size due to allometry (Zelditch et al., 2004). Where appropriate, we transformed response variables of ANOVA/MANOVA analyses to ensure normality (‘bestNormalize’ package in R: Peterson, 2017): scaled centroid size (Box‐Cox: inbreeding data); RW 1–5 (Yeo Johnson: inbreeding and diet data); leg length (orderNorm: inbreeding data). Model residuals were checked for normality with the ‘shapiro.test’ functon in R. All models were simplified by removing non‐significant terms (*p* < .05).

The inbreeding depression assay had a paired design (i.e. families were paired to form groups) and therefore we also calculated mean scaled centroid size, RW 1–5 scores, and leg length for inbred and outbred individuals at the group level (*n* = 53 groups; see Figure 1) and compared them with paired *t*‐tests (Roff, 1998). Only leg length required transformation (orderNorm) in these analyses.

We assessed the extent of genetic and environment‐induced condition dependence of traits by calculating effect sizes for inbreeding and diet treatments. For ANOVA/MANOVA analyses we calculated the partial Eta^2^ statistic (‘rstatix’ package in R for ANOVA: Kassambara, 2019; ‘heplots’ package in R for MANOVA: Fox et al., 2009), which measures the proportion of variance in the trait explained by inbreeding or diet treatments. For paired *t*‐tests, we used the Cohen's *d* statistic (‘rstatix’ package in R: Kassambara, 2019), which calculates the difference between inbred/outbred group means of traits divided by the pooled standard deviation. All statistical analyses were performed using R software (version 3.6.3; R Core Team, 2020), unless stated otherwise.

## 3 RESULTS

### 3.1 Inbreeding effects

**FIGURE 3:**
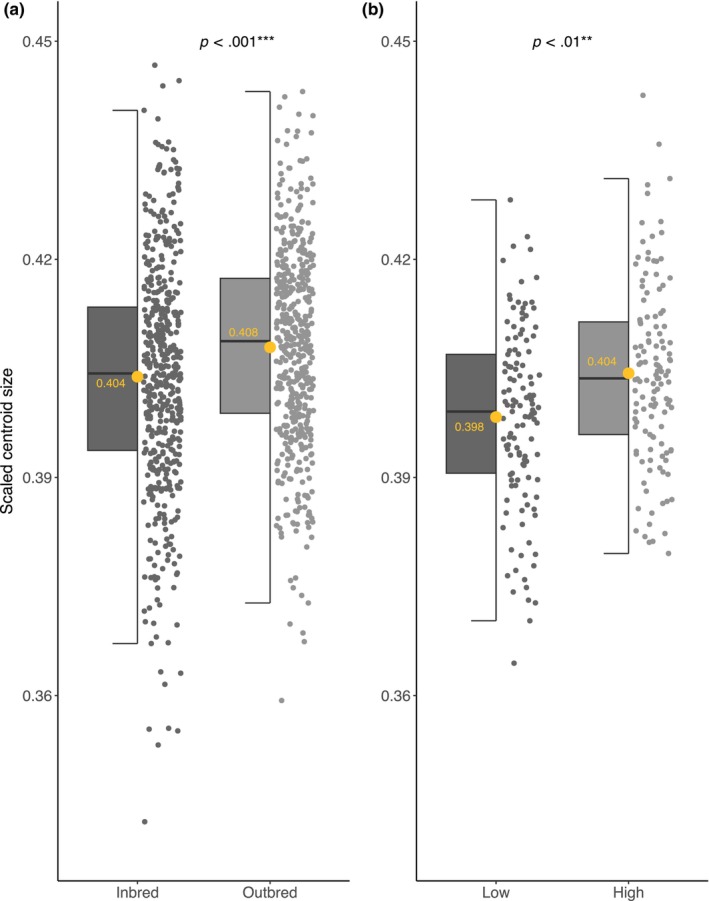
Effect of treatment on genital size. Panel (a) shows that inbred males had significantly smaller genital arches compared to outbred males and (b) shows males on a restricted diet (low condition) had significantly smaller genital arches compared to males on an ad‐libitum diet (high condition). Raw data are represented as grey points and boxplots show median values, upper/lower quartiles and whiskers representing 1.5* the interquartile range. Means for each treatment are represented as yellow points and their corresponding values are shown as yellow text.

There was significant inbreeding depression for genital size (controlling for body size: leg length as a covariate). When inbred individuals were compared with outbred as a whole (i.e. regardless of the group structure to the data), scaled centroid size of outbred individuals ($\bar{x}$ = 0.408, SE = 0.001, *n* = 489) was significantly greater than that of the inbred individuals ($\bar{x}$ = 0.404, SE = 0.001, *n* = 487; *p* < .001; Table 2 and see Table S2 for full model; Figure 3). Paired analysis confirmed this finding (Table 3). Examination of partial Eta^2^ and Cohen's *d* statistics also revealed appreciable effect sizes (partial η^2^ > 0.01; Cohen's *d* > 0.2) for inbreeding impacts on genital size in both the ANOVA (Table 2) and pairwise analysis (Table 3).

**TABLE 2:** Results of ANOVA and MANOVA analyses examining effects of (a) inbreeding and (b) diet on scaled centroid size (SCS), genital shape (RW 1–5) and leg length (body size).

Analysis	Trait	*F*_df_	*p‐value*	Partial Eta^2^
(a) Inbreeding effects				
ANOVA	SCS	16.83_1973_	<.001***	0.020
ANOVA	Leg length	2.71_1974_	.100	0.003
MANOVA	RW 1–5	1.56_9964_	.170	0.008
(b) Diet effects				
ANOVA	SCS	8.97_1247_	<.01**	0.035
ANOVA	Leg length	19.57_1248_	<.001***	0.073
MANOVA	RW 1–5	2.52_9239_	.030*	0.049
ANOVA	RW 1	1.37_1247_	.243	0.006
ANOVA	RW 2	0.27_1247_	.018*	0.023
ANOVA	RW 3	1.23_1247_	.269	0.005
ANOVA	RW 4	3.95_1247_	.048*	0.016
ANOVA	RW 5	0.11_1247_	.738	<0.001

*Note*: Significant *p* values are denoted by asterisks. As diet had a significant influence on the multivariate combination of RW 1–5, post‐hoc univariate ANOVAs were carried out to show which warps generated the multivariate significance. Effect sizes are reported as partial Eta^2^ estimates. Full models, including covariates, can be found in Table S2.

**TABLE 3:** Results from the pairwise comparisons of mean scaled centroid size (SCS), mean leg length (body size), and mean RW 1–5 scores between inbred and outbred individuals.

	Inbred		Outbred		*t*_52_	*p‐value*	Cohen's *d*
	$\bar{x}$ value	SE	$\bar{x}$ value	SE			
SCS	0.404	0.001	0.408	0.001	−3.07	**.003**	**0.421**
Leg length	513.366	1.610	515.052	1.503	−1.80 × 10^−17^	1.000	<0.001
RW 1	9.50 × 10^−3^	2.55 × 10^−3^	−1.90 × 10^−3^	2.85 × 10^−3^	0.86	.395	0.117
RW 2	4.90 × 10^−5^	2.00 × 10^−3^	−3.61 × 10^−**4**^	1.41 × 10^−3^	0.21	.835	0.029
RW 3	−1.56 × 10^−4^	1.55 × 10^−3^	−5.10 × 10^−4^	1.42 × 10^−3^	0.21	.837	0.028
RW 4	6.07 × 10^−4^	1.24 × 10^−3^	−1.44 × 10^−4^	1.45 × 10^−3^	0.48	.635	0.066
RW 5	−2.10 × 10^−4^	9.44 × 10^−4^	−5.02 × 10^−4^	9.83 × 10^−4^	0.26	.797	0.036

*Note*: Effect sizes are reported as Cohen's *d* estimates. Outbred males had a significantly larger scaled centroid size than inbred males; however, there were no significant differences for leg length or RWs 1–5. Scaled centroid size, leg length, and RW scores for inbred and outbred specimens were averaged within replicate lines and then across replicates for each group (*n* = 53) before comparisons. The significant SCS result remains with Bonferroni correction (critical *p* = .007) represented in bold.

**FIGURE 4:**
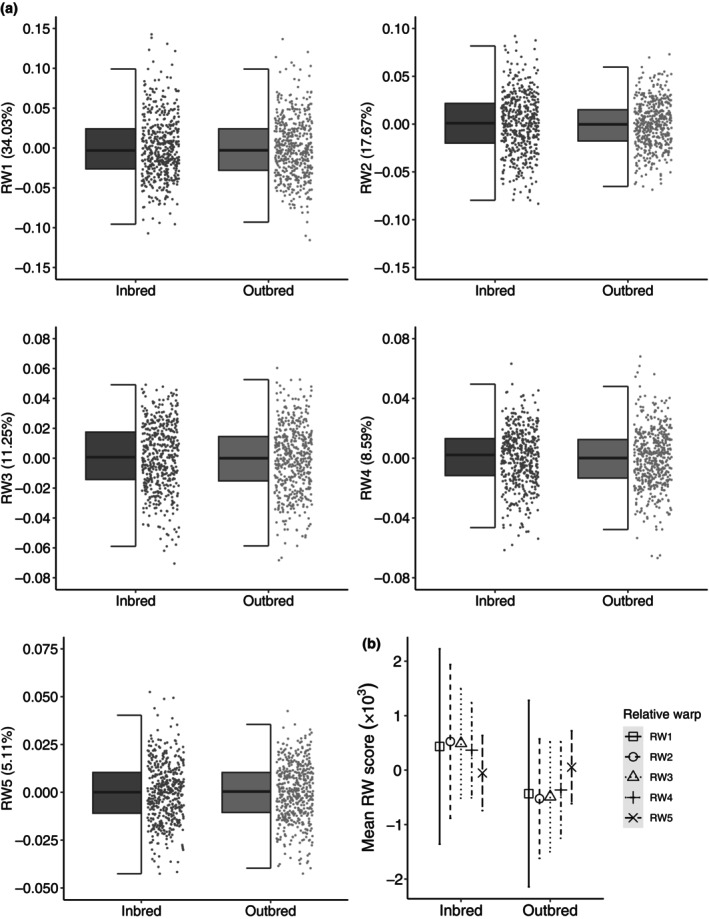
The lack of effect of inbreeding on genital shape (see Figure 2 for the inbreeding experiment consensus shape). Panel (a) shows Relative Warp (RW) scores for RW 1–5 for inbred and outbred males, where raw data are represented as grey points and boxplots show median values, upper/lower quartiles and whiskers representing 1.5* the interquartile range. There was no significant effect of inbreeding on the multivariate combination of RW 1–5 (*p* = .17) and therefore univariate tests on each RW were not carried out. For ease of visual comparisons, (b) shows mean RW scores for RW 1–5 (represented by different symbols) and SE ±1 (represented by error bars). Data has been scaled ×1000 for visual clarity.

**FIGURE 5:**
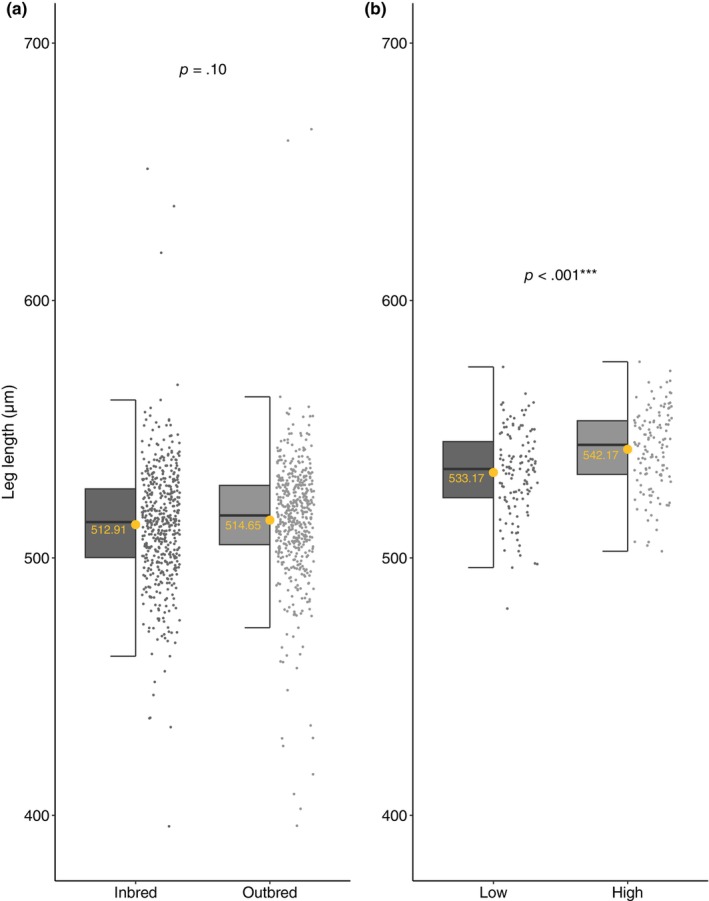
Effect of treatment on leg length (body size). Panel (a) shows no significant difference in leg length between inbred and outbred males and (b) shows that males on a restricted diet (low condition) had significantly shorter leg length compared to males on an ad‐libitum diet (high condition). Raw data are represented as grey points and boxplots show median values, upper/lower quartiles and whiskers representing 1.5* the interquartile range. Means for each treatment are represented as yellow points and their corresponding values are shown as yellow text.

RWs 1–5 collectively explained 76.65% of the variance in shape, but inbreeding status did not significantly influence genital shape (controlling for body size: leg length as a covariate), as represented by the multivariate combination of RWs 1–5 (*p* = .170; Table 2 and see Table S2 for full model; Figure 4). Similarly, inbreeding had no effect on leg length, our index of body size (*p* = .100; Table 2; Figure 5a). Paired analysis also found no significant difference in mean RW scores or leg length (body size) for inbred and outbred specimens across all 5 RWs' (Table 2). Effect sizes were negligible (partial η^2^ < 0.01; Cohen's *d* < 0.2) for the effects of inbreeding on RW scores and leg length, which is in agreement with the ANOVA/MANOVA (see partial Eta^2^ in Table 2) and pairwise analyses (see Cohen's *d* in Table 3). These findings suggest that neither genital shape nor leg length suffer from significant inbreeding depression.

### 3.2 Diet effects

**FIGURE 6:**
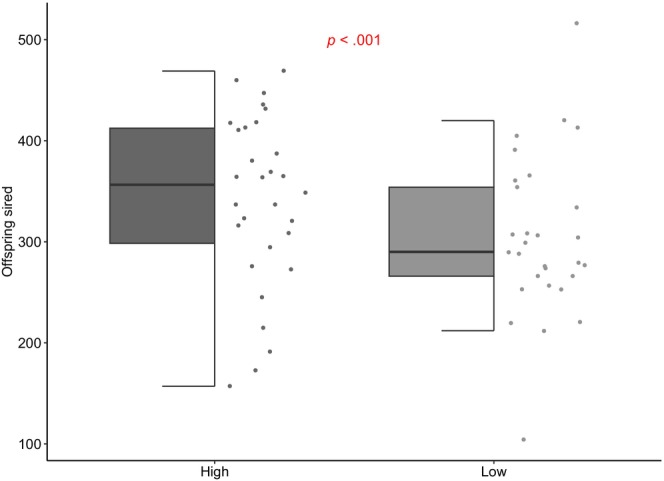
The effect of diet on male fitness. Grey points show the total offspring sired by males on the restricted (low condition) or ad‐libitum (high condition) diet. Boxplots show median values, upper/lower quartiles and whiskers representing 1.5* the interquartile range.

Mean male reproductive output significantly differed between diet treatments, with diet‐restricted males siring on average 11% fewer offspring (Mean ± SE offspring: ad‐libitum food group = 341.67 ± 3.4; diet‐restricted group = 304.14 ± 3.3; GlzM χ^2^(1) = 64.18, *n* = 30; *p* < .001; Figure 6). Therefore the diet restriction treatment reduced male condition, as these males were of lower fitness.

**FIGURE 7:**
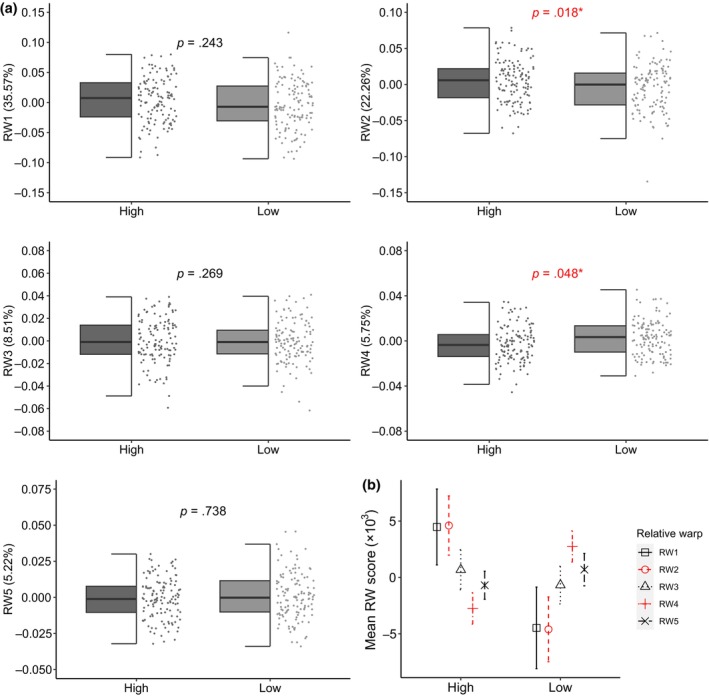
The effect of diet on genital shape. Panel (a) shows Relative Warp (RW) scores for RW 1–5 for males on a restricted (low condition) or ad‐libitum (high condition) diet, where raw data are represented as grey points and boxplots show median values, upper/lower quartiles and whiskers representing 1.5* the interquartile range. There was a significant effect of inbreeding on the multivariate combination of RW 1–5 (*p* = .030) and therefore univariate tests on each RW were carried out (shown as *p* values above each plot). Both RW2 and RW4 were significant. For ease of visual comparisons, (b) shows mean RW scores for RW 1–5 (represented by different symbols) and SE ±1 (represented by error bars). Data has been scaled ×1000 for visual clarity.

**FIGURE 8:**
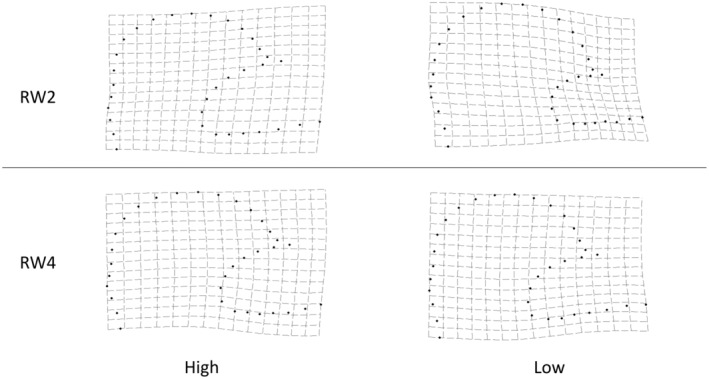
Genital arch shape changes associated with diet. Both RW2 and RW4 were significantly impacted by diet. Thin plate splines demonstrate shape changes associated with the most extreme Relative Warp scores for males on a restricted (low condition) or ad‐libitum (high condition) diet.

Genital size was dependent on diet (controlling for body size: leg length as a covariate), with larger genitals found in males from the ad‐libitum food (high condition) treatment (high condition $\bar{x}$ = 0.404, SE = 0.001, *n* = 125; low condition $\bar{x}$ = 0.398, SE = 0.001, *n* = 125; *p* < .01; Table 2 and see Table S2 for full model; Figure 3b). Genital shape was also influenced by diet (controlling for body size: leg length as a covariate; *p* = .030; Table 2 and see Table S2 for full model; Figures 7 and 8). Here RWs 1–5 cumulatively explained 77.31% of the shape variance and both diet and scaled centroid size influenced the multivariate combination of RWs (see Table S2). Post‐hoc univariate tests revealed the multivariate effect was driven by RW 2 and RW 4 (Table 2 and see Table S2 for full model), with males from the high condition treatment having significantly higher scores for warp 2 and marginally significantly lower scores for warp 4 (Figures 7 and 8). Increasing scores in high condition males along RW 2 are associated with compression of the posterior lobes anterior section, leading to an increase in the ventral cup aperture (Figure 8). Decreases in RW 2 scores in low condition males show the opposite shape changes, with posterior–anterior lobe elongation, resulting in dorso‐ventral compression in the ventral cup (Figure 8). Decreasing scores in high condition males along RW 4 are associated with downward curvature of the basal section of the ventral cup, resulting in an increase in the ventral cup aperture (Figure 8). Increases in RW 4 scores show the opposite shape changes, with straightening of the basal section of the ventral cup, decreasing the aperture of the ventral cup (Figure 8). We also found a significant effect of diet on leg length (body size), with larger leg length found in high condition males (high condition $\bar{x}$ = 542.170, SE = 1.397, *n* = 125; low condition $\bar{x}$ = 533.173, SE = 1.478, *n* = 125; *p* < .001; Table 2; Figure 5b). Examination of partial Eta^2^ statistics revealed appreciable effect sizes (partial η^2^ > 0.01) for the diet manipulation on all three traits (Table 2).

## 4 DISCUSSION

Our major findings were that genital size and shape were impacted by dietary manipulation, and genital size, but not genital shape, showed inbreeding depression. Thus, male genitals showed condition dependence, but effects manifested depended on how condition was manipulated. As we discuss below, these results also imply (i) different components of genital morphology are subject to different forms of selection or are differently linked to fitness and (ii) genitals can be condition‐dependent much like other sexually selected characters.

### 4.1 Condition and genital size

Many sexually selected traits are condition‐dependent (Bonduriansky & Rowe, 2005; Cotton, Fowler, & Pomiankowaki, 2004; Delcourt & Rundle, 2011; reviews in Andersson, 1994; Cotton et al., 2006; Hosken & House, 2011), and consistent with this, we found that inbred individuals (those in poor genetic condition: Okada et al., 2011; Townsend et al., 2010) had smaller genital arches, while outbred individuals (those in good genetic condition) had larger arches (controlling for body size). Similarly, when condition was altered through diet manipulation, well‐fed, good condition individuals had larger genital arches (controlling for body size) compared to poor condition individuals reared on a restricted diet. In general, little is known about the effects of condition on male genitals, but sperm, another primary sexual trait, is impacted by inbreeding (Asa et al., 2007; Fitzpatrick & Evans, 2009; Gage et al., 2006; Gomendio et al., 2000). Environment‐induced condition dependence of genital size has also been found in water striders (Arnqvist & Thornhill, 1998). However, other studies (*D. melanogaster*: Shingleton et al., 2009; *Onthophagus taurus*: House & Simmons, 2007; *Callosobruchus maculatus*: Cayetano & Bonduriansky, 2015) reported no condition dependence for male genitals. It is not clear why these differences exist, but the contrasting results of our study and that for *D. melanogaster* (Shingleton et al., 2009) may be due to the powerful geometric morphometric approach we employed here.

Inbreeding depression for arch size also provides information about past selection likely to have acted on size. Specifically, traits that have been subjected to directional selection are expected to show inbreeding depression, whereas those under stabilising selection (or weakly linked to fitness) will not (Lynch & Walsh, 1998). Genital form in *D. simulans* directly influences male fitness (House et al., 2021; also see Jagadeeshan & Singh, 2006) and evolves through sexual selection during experimental evolution (House et al., 2013). That is, form is linked to fitness. It therefore appears that genitals have been under directional selection for increased arch size. This inference can be made because inbreeding depression is always away from the direction of highest fitness (Lynch & Walsh, 1998) and inbred individuals had smaller genitals. Consistent with this inference, directional selection for larger intromittent organs has been documented in other taxa (Langerhans et al., 2005; and see Kahn et al., 2010) and inbreeding depression for sexually selected characters has been documented more broadly (e.g. Ketola & Kotiaho, 2010; Okada et al., 2011; Reid et al., 2011; Sheridan & Pomiankowski, 1997). Episodes of directional selection are thought to be responsible for the rapid divergence of genital form seen across closely related species (Eberhard, 1985; Jagadeeshan & Singh, 2006), including in *Drosophila* (Zeng et al., 2000). Interestingly, a recent study that quantified the strength and form of selection on *D. simulans* genitals, indicates that genital arch size seems to be currently under stabilising selection (House et al., 2021). Collectively, this information suggests selection on genital size has been directional (possibly during species divergence: see Coyne, 1983; Eberhard, 1983), but selection on size may now be stabilising, consistent with the “one‐size‐fits‐all” hypothesis (Eberhard et al., 1998).

It should be noted that inbreeding manipulations may also impact resource acquisition, assimilation and allocation to traits (Rowe & Houle, 1996). However, these potential effects are likely to be masked by the ad‐libitum diet we provided in our inbreeding treatment (for studies examining effects of genetic by environment interactions see: Bonduriansky et al., 2015; Cotton, Fowler, & Pomiankowski, 2004; de Boer et al., 2018; Hooper & Bonduriansky, 2022; Howie et al., 2019; Vega‐Trejo et al., 2018). Indeed, we also found that our measure of body size (leg length) was not impacted by inbreeding, which is consistent with resource acquisition being unaffected by inbreeding (see Section 4.3). Therefore, the effects of inbreeding on genital size most likely reflect the degree of directional dominance and its direct impacts on the trait.

### 4.2 Condition and genital shape

The effects of condition on genital shape depended on the manipulation employed. There was no impact of inbreeding on genital shape, but diet restriction had an effect (again controlling for body size). The diet results therefore indicate that genital shape can be condition‐dependent, while lack of inbreeding depression suggests that shape is either weakly linked to fitness or has been under stabilising selection. It seems likely to us that genital shape is linked to fitness. Firstly, the shape of the genital arch greatly differs between *D. simulans* and its close relatives (Coyne, 1983; Jagadeeshan & Singh, 2006) and these observed interspecific differences are unlikely to be explained by drift because of large population sizes in these taxa (Eyre‐Walker et al., 2002). There is also direct evidence that genital shape influences male fitness (e.g. in *D. simulans*: House et al., 2013; House et al., 2021; and *D. sechellia*: Frazee et al., 2021). House et al. (2021) also found that elements of genital shape are currently under stabilising selection in *D. simulans*. Similar to genital size, stabilising selection on shape may be generated by the “one‐size‐fits‐all” mechanism (Eberhard et al., 1998), but it may also arise from opposing natural and sexual selection (e.g. House et al., 2013), providing a potential explanation for the lack of inbreeding depression in shape.

Lack of inbreeding depression in sexually selected traits has been reported previously, but the implications of this were not always appreciated. For example, attributes of sperm and testis form showed no inbreeding depression in sticklebacks, which may have resulted from low sample size (Mehlis et al., 2012; but see Ala‐Honkola et al., 2013). However, this lack of inbreeding depression could also be indicative of stabilising selection on these characters, especially since testes and sperm form are typically linked to male fitness (e.g. Gage et al., 2004; Hosken & Ward, 2001; Pitnick et al., 2001; Reviewed in Birkhead et al., 2009). Other studies have also reported stabilising selection on sexual traits, including cuticular hydrocarbons (CHCs) in *D. serrata* (Rundle & Chenoweth, 2011; and also see Ingleby et al., 2014 and Wheeler et al., 2012), and genitals in *D. simulans* (House et al., 2021) and other arthropods (e.g. House et al., 2020; Wojcieszek & Simmons, 2012).

### 4.3 Condition and leg size

We found no evidence of inbreeding depression for leg size, but our diet manipulation led to males on diet restriction having smaller legs. This latter effect is no surprise given that condition‐dependent growth is well documented (e.g. House & Simmons, 2007; Hunt & Simmons, 1997) and *Drosophila* species have lower growth rates and smaller adult size with resource limitation (and see Blanckenhorn, 1999; Dmitriew, 2011). The lack of an inbreeding effect is consistent with historical stabilising selection on body size, which is expected for morphological traits (Mackay, 2010) and is in agreement with previous work on *Drosophila* body size (Lefranc & Bundgaard, 2000). Additionally, these results for leg size are consistent with some of our findings for wing size (Okada et al., 2011; also see Wright et al., 2008).

## 5 CONCLUSIONS

To conclude, our major finding was that genitals can be condition‐dependent, but evidence for this was contingent upon the means of the condition manipulation. Our study also highlights how lack of inbreeding depression for a trait does not automatically equate to a lack of condition dependence. Rather, inbreeding effects reflect directional dominance for a character and not just condition.

Finally, the condition dependence of genital characters (size/shape) has received relatively little attention, but our results indicate male genitals can be like many other sexual traits in this regard. The mechanistic function of the genital arches needs to be investigated further to assess how differences in genital size and shape generate the variation in male fitness that is implied by these and other (e.g. House et al., 2021) findings, and assessment of the genetic and environmental variance in female genitals is needed to assess the veracity of some of our inferences.

## AUTHOR CONTRIBUTIONS

**Tanya M. Pennell:** Formal analysis (equal); writing – original draft (lead); writing – review and editing (equal). **Manmohan D. Sharma:** Conceptualization (equal); data curation (equal); formal analysis (equal); investigation (equal); methodology (equal). **Andreas Sutter:** Data curation (supporting). **Drew T. Wilson:** Investigation (equal); Data curation (supporting). **Clarissa M. House:** Data curation (supporting); writing – reviewing and editing equal. **David J. Hosken:** Conceptualization (equal); funding acquisition (equal); investigation (equal); methodology (equal); supervision (equal); writing – original draft (equal); writing – review and editing (equal).

## FUNDING INFORMATION

This work was supported by Natural Environment Research Council (NE/G005303/1 – DJH).

## Supporting information


Data S1.

Table S1.

Table S2.

## Data Availability

Analyses reported in this article can be reproduced using data published in the Dryad repository http://doi.org/10.5061/dryad.rn8pk0pk9.
