# Supplementary material for: The condition‐dependence of male genital size and shape
Source: Ecol Evol. 2024 Mar 17;14(3):e11180. doi: 10.1002/ece3.11180 (PMC10944674; doi:10.1002/ece3.11180)
Supplement: Supplementary file 2 — Table S1. [file ECE3-14-e11180-s003.docx]

**Table S1.** **Diet manipulation recipe.**

Food Type AL (*ad-libitum*) is a standard porridge mix which was used to maintain the stock population and generate male experimental flies and DR is the diet restriction treatment. All recipes make 1000ml of medium after cooking for 10 minutes.

| **Food Type** | **Diet Restriction (%)** | **Brown Sugar (g)** | **Maize Meal (g)** | **Brewer’s Yeast (g)** | **Water (ml)** | **Agar (g)** | **Nipagin (g)** | **Propionic Acid (ml)** |
| --- | --- | --- | --- | --- | --- | --- | --- | --- |
| **AL** | 0 | 113 | 80 | 27 | 1200 | 12 | 2 | 6 |
| **DR** | 40 | 68 | 48 | 16 | 1200 | 12 | 2 | 6 |
